# 2-(6-Methyl-2-pyrid­yl)-1,1-diphenyl­ethanol

**DOI:** 10.1107/S1600536808025026

**Published:** 2008-08-09

**Authors:** Wei-Jin Gu, Bing-Xiang Wang

**Affiliations:** a Department of Applied Chemistry, Nanjing Normal University, Nanjing 210097, People’s Republic of China

## Abstract

The title compound, C_20_H_19_NO, was prepared from 2,6-lutidine and benzophenone. There are two symmetry-independent mol­ecules in the asymmetric unit. Each mol­ecule is involved in one intra­molecular O—H⋯N hydrogen bond. In the crystal structure, helical chains are formed along the *b* axis by weak π–π inter­actions between neighbouring mol­ecules [centroid–centroid distances between the pyridyl rings of the two independent mol­ecules = 4.041 (3) and 4.051 (3) Å].

## Related literature

For related literature, see: Berg & Holm (1985 ▶); Dehnicke *et al.* (2001 ▶); Gibson *et al.* (2007 ▶); Koning *et al.* (2000 ▶); Yip *et al.* (2003 ▶).
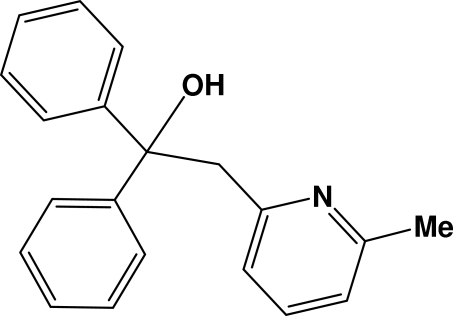


## Experimental

### 

#### Crystal data

C_20_H_19_NO
*M* *_r_* = 289.36
Monoclinic, 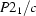

*a* = 13.466 (2) Å
*b* = 8.022 (1) Å
*c* = 30.220 (3) Å
β = 102.874 (3)°
*V* = 3182.4 (7) Å^3^
*Z* = 8
Mo *K*α radiation
μ = 0.07 mm^−1^
*T* = 291 (2) K
0.30 × 0.26 × 0.24 mm

#### Data collection

Bruker SMART APEX CCD diffractometer
Absorption correction: multi-scan (*SADABS*; Bruker, 2000 ▶) *T*_min_ = 0.98, *T*_max_ = 0.98
24109 measured reflections
6247 independent reflections
4901 reflections with *I* > 2σ(*I*)
*R*_int_ = 0.039

#### Refinement

*R*[*F* ^2^ > 2σ(*F* ^2^)] = 0.049
*wR*(*F* ^2^) = 0.116
*S* = 1.02
6247 reflections
405 parameters
H atoms treated by a mixture of independent and constrained refinement
Δρ_max_ = 0.12 e Å^−3^
Δρ_min_ = −0.12 e Å^−3^

### 

Data collection: *SMART* (Bruker, 2000 ▶); cell refinement: *SMART*; data reduction: *SAINT* (Bruker, 2000 ▶); program(s) used to solve structure: *SHELXTL* (Sheldrick, 2008 ▶); program(s) used to refine structure: *SHELXTL*; molecular graphics: *SHELXTL*; software used to prepare material for publication: *SHELXTL*.

## Supplementary Material

Crystal structure: contains datablocks global, I. DOI: 10.1107/S1600536808025026/im2078sup1.cif

Structure factors: contains datablocks I. DOI: 10.1107/S1600536808025026/im2078Isup2.hkl

Additional supplementary materials:  crystallographic information; 3D view; checkCIF report

## Figures and Tables

**Table 1:** Hydrogen-bond geometry (Å, °)

*D*—H⋯*A*	*D*—H	H⋯*A*	*D*⋯*A*	*D*—H⋯*A*
O1—H1*A*⋯N1	0.96 (2)	1.89 (2)	2.724 (2)	144 (2)
O2—H2*A*⋯N2	0.96 (2)	1.92 (2)	2.718 (2)	139 (2)
C18—H18⋯O2^i^	0.93	2.70	3.518 (2)	147
C38—H38⋯O1^ii^	0.93	2.65	3.472 (2)	147

Symmetry codes: (i) 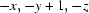; (ii) 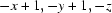.

**Fig. 1.**
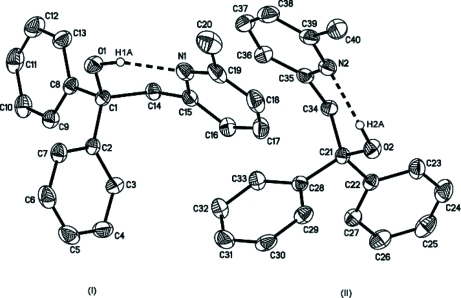
A view of the title compound showing the atom-numbering scheme and displacement ellipsoids drawn at 30% probability level. Dashed lines indicate hydrogen bonds and all H atoms except those involved in hydrogen bonding have been omitted for clarity.

**Fig. 2.**
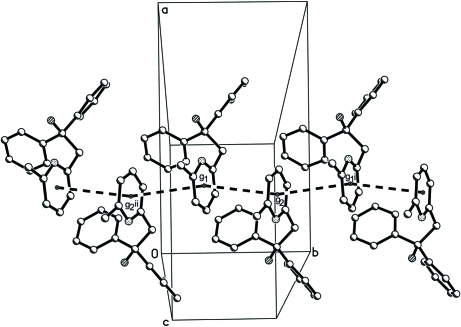
A view of the helical chain-like structure along the b axis. (i: x,1 + y,z; ii:x,-1 + y,z).

## supplementary crystallographic information

### Comment

There is a growing interest in the chemistry of metal-nitrido complexes, particularly those of late transition metals (Dehnicke *et al.*, 2001). 2-(6-Methyl-pyridin-2-yl)-1,1-diphenyl-ethanol has been used as a multianionic chelating (N, O) ligand and coordinated with transition metals and main group elements, such as Ru, Os, Mo, B, Si *etc*. (Yip *et al.*, 2003; Gibson *et al.*, 2007). It can be prepared from 2,6-lutidine in moderate yield (Koning *et al.*, 2000). During our studies, we obtained single crystals of the title compound report its crystal structure herein.

The crystal structure of title compound, C_20_H_19_NO, shows that all the bond lengths and angles have normal values. In an asymmetric unit there are two symmetry independent molecules I and II. Each molecule has one intramolecular O—H···N hydrogen bond (O1—H1A···N1, O2—H2A···N2)(Fig. 1).

Molecule I exhibits two benzene rings A (C2—C7) and B (C8—C13) as well as a pyridine ring C (N1/C15—C19) that are not coplanar with respect to each other. The dihedral angles between rings A and B, B and C, and C and A measure to 82.02 (6)°, 47.06 (6)°, and 85.87 (5)°, respectively.

Molecule II looks pretty much the same as molecule I, but the dihedral angles are significantly different. The angles between rings D(C22—C27) and E(C28—C33), E and F(N2/C35—C39), and F and D are 83.58 (6)°, 85.44 (5)°, and 49.70 (6)°, respectively.

In the crystal packing weak π–π interactions between neighbouring molecules I and II are observed, the distance of g1-g2 being 4.041 (3) and 4.051 (3) Å (g1 is center of mass of N1/C15—C19, g2 is center of mass of N2/C35—C39). Helical chains along the *b* axis are formed by these interactions (Fig. 2).

The additional weak intermolecular C18—H18···O2^iii^ and C38—H38···O1^iv^ (iii: -*x*,1 - *y*,-*z*; iv: 1 - *x*,1 - *y*,-*z*) hydrogen bonds play part an important role linking the helical chains to form the three-dimensional crystal structure.

### Experimental

2-(6-Methyl-pyridin-2-yl)-1,1-diphenyl-ethanol was prepared from 2,6-lutidine and benzophenone according to a procedure described in the literature (Berg & Holm, 1985, yield: 60%). Colorless crystals were obtained by recrystallization from light petroleum-ethyl acetate(vol. ratio: 5:1) at room temperature.

^1^*H*-NMR (CDCl_3_, 400 MHz): δ = 8.1 (1 H, s, OH), 6.8–7.5 (13 H, 2 Ph + 3H), 3.7 (2 H, s, CH_2_), 2.5 (3 H, s, CH_3_).

### Refinement

The H atoms were placed in calculated positions except O—H atoms and included as part of a riding model, with C—H = 0.93–0.97 Å, and with U_equiv_ values set at 1.2–1.5 U_equiv_ of the parent atoms. The O—H atoms were located in the Fourier difference map and refined with a given isotropic thermal parameters 1.2 times the U_equiv_ for the parent atom.

### Crystal data

**Table:**

C_20_H_19_NO	*F*_000_ = 1232
*M**_r_* = 289.36	*D*_x_ = 1.208 Mg m^−^^3^
Monoclinic, *P*2_1_/*c*	Mo *K*α radiation λ = 0.71073 Å
Hall symbol: -P 2ybc	Cell parameters from 9992 reflections
*a* = 13.466 (2) Å	θ = 2.3–27.7º
*b* = 8.022 (1) Å	µ = 0.07 mm^−^^1^
*c* = 30.220 (3) Å	*T* = 291 (2) K
β = 102.874 (3)º	Bloc, colourless
*V* = 3182.4 (7) Å^3^	0.30 × 0.26 × 0.24 mm
*Z* = 8	

### Data collection

**Table:**

Bruker SMART APEX CCD diffractometer	6247 independent reflections
Radiation source: sealed tube	4901 reflections with *I* > 2σ(*I*)
Monochromator: graphite	*R*_int_ = 0.039
*T* = 291(2) K	θ_max_ = 26.0º
φ and ω scans	θ_min_ = 1.6º
Absorption correction: multi-scan(SADABS; Bruker, 2000)	*h* = −16→16
*T*_min_ = 0.98, *T*_max_ = 0.98	*k* = −9→9
24109 measured reflections	*l* = −36→37

### Refinement

**Table:**

Refinement on *F*^2^	Secondary atom site location: difference Fourier map
Least-squares matrix: full	Hydrogen site location: inferred from neighbouring sites
*R*[*F*^2^ > 2σ(*F*^2^)] = 0.049	H atoms treated by a mixture of independent and constrained refinement
*wR*(*F*^2^) = 0.116	*w* = 1/[σ^2^(*F*_o_^2^) + (0.05*P*)^2^ + 0.55*P*] where *P* = (*F*_o_^2^ + 2*F*_c_^2^)/3
*S* = 1.03	(Δ/σ)_max_ < 0.001
6247 reflections	Δρ_max_ = 0.12 e Å^−^^3^
405 parameters	Δρ_min_ = −0.12 e Å^−^^3^
Primary atom site location: structure-invariant direct methods	Extinction correction: none

### Special details

**Table:**

Geometry. All e.s.d.'s (except the e.s.d. in the dihedral angle between two l.s. planes) are estimated using the full covariance matrix. The cell e.s.d.'s are taken into account individually in the estimation of e.s.d.'s in distances, angles and torsion angles; correlations between e.s.d.'s in cell parameters are only used when they are defined by crystal symmetry. An approximate (isotropic) treatment of cell e.s.d.'s is used for estimating e.s.d.'s involving l.s. planes.Least-squares planes (*x*,*y*,*z* in crystal coordinates) and deviations from them (* indicates atom used to define plane)7.9264 (0.0081) *x* + 2.5384 (0.0055) *y* + 17.9519 (0.0173) *z* = 7.2409 (0.0023)* 0.0041 (0.0011) C2 * -0.0032 (0.0012) C3 * 0.0016 (0.0013) C4 * -0.0007 (0.0013) C5 * 0.0017 (0.0013) C6 * -0.0033 (0.0012) C7Rms deviation of fitted atoms = 0.00278.6157 (0.0075) *x* - 6.1646 (0.0038) *y* - 4.0922 (0.0231) *z* = 2.4746 (0.0072)Angle to previous plane (with approximate e.s.d.) = 82.02 (0.06)* -0.0038 (0.0011) C8 * 0.0059 (0.0011) C9 * -0.0038 (0.0013) C10 * -0.0004 (0.0014) C11 * 0.0025 (0.0014) C12 * -0.0004 (0.0012) C13Rms deviation of fitted atoms = 0.0034- 0.0031 (0.0098) *x* + 7.0734 (0.0030) *y* - 13.8930 (0.0198) *z* = 1.4744 (0.0034)Angle to previous plane (with approximate e.s.d.) = 47.06 (0.06)* 0.0019 (0.0011) N1 * 0.0034 (0.0011) C15 * -0.0039 (0.0012) C16 * -0.0005 (0.0014) C17 * 0.0056 (0.0014) C18 * -0.0064 (0.0012) C19Rms deviation of fitted atoms = 0.00417.9264 (0.0081) *x* + 2.5384 (0.0055) *y* + 17.9519 (0.0173) *z* = 7.2409 (0.0023)Angle to previous plane (with approximate e.s.d.) = 85.87 (0.05)* 0.0041 (0.0011) C2 * -0.0032 (0.0012) C3 * 0.0016 (0.0013) C4 * -0.0007 (0.0013) C5 * 0.0017 (0.0013) C6 * -0.0033 (0.0012) C7Rms deviation of fitted atoms = 0.00278.6502 (0.0076) *x* + 6.1357 (0.0039) *y* - 2.9169 (0.0234) *z* = 5.1262 (0.0059)Angle to previous plane (with approximate e.s.d.) = 49.02 (0.07)* -0.0040 (0.0011) C22 * 0.0029 (0.0013) C23 * -0.0003 (0.0014) C24 * -0.0010 (0.0014) C25 * -0.0002 (0.0013) C26 * 0.0028 (0.0012) C27Rms deviation of fitted atoms = 0.0024- 8.0302 (0.0080) *x* + 2.4726 (0.0054) *y* + 25.8511 (0.0117) *z* = 4.4687 (0.0039)Angle to previous plane (with approximate e.s.d.) = 83.58 (0.06)* 0.0063 (0.0011) C28 * -0.0023 (0.0012) C29 * -0.0051 (0.0013) C30 * 0.0085 (0.0014) C31 * -0.0043 (0.0013) C32 * -0.0032 (0.0012) C33Rms deviation of fitted atoms = 0.0053- 0.0603 (0.0094) *x* + 7.0521 (0.0030) *y* - 14.0087 (0.0195) *z* = 4.9759 (0.0037)Angle to previous plane (with approximate e.s.d.) = 85.44 (0.05)* -0.0024 (0.0011) N2 * 0.0054 (0.0011) C35 * -0.0025 (0.0012) C36 * -0.0031 (0.0013) C37 * 0.0060 (0.0013) C38 * -0.0034 (0.0012) C39Rms deviation of fitted atoms = 0.00408.6502 (0.0076) *x* + 6.1357 (0.0039) *y* - 2.9169 (0.0234) *z* = 5.1262 (0.0059)Angle to previous plane (with approximate e.s.d.) = 49.70 (0.06)* -0.0040 (0.0011) C22 * 0.0029 (0.0013) C23 * -0.0003 (0.0014) C24 * -0.0010 (0.0014) C25 * -0.0002 (0.0013) C26 * 0.0028 (0.0012) C27Rms deviation of fitted atoms = 0.0024
Refinement. Refinement of *F*^2^ against ALL reflections. The weighted *R*-factor *wR* and goodness of fit *S* are based on *F*^2^, conventional *R*-factors *R* are based on *F*, with *F* set to zero for negative *F*^2^. The threshold expression of *F*^2^ > σ(*F*^2^) is used only for calculating *R*-factors(gt) *etc*. and is not relevant to the choice of reflections for refinement. *R*-factors based on *F*^2^ are statistically about twice as large as those based on *F*, and *R*- factors based on ALL data will be even larger.

### Fractional atomic coordinates and isotropic or equivalent isotropic displacement parameters (Å^2^)

**Table:**

	*x*	*y*	*z*	*U*_iso_*/*U*_eq_	
C1	0.55525 (11)	0.30363 (18)	0.12015 (5)	0.0333 (3)	
C2	0.52945 (10)	0.15683 (18)	0.14763 (5)	0.0333 (3)	
C3	0.46070 (12)	0.1698 (2)	0.17575 (5)	0.0432 (4)	
H3	0.4257	0.2692	0.1767	0.052*	
C4	0.44374 (14)	0.0364 (3)	0.20236 (6)	0.0552 (5)	
H4	0.3981	0.0474	0.2212	0.066*	
C5	0.49408 (16)	−0.1119 (3)	0.20098 (7)	0.0631 (5)	
H5	0.4823	−0.2014	0.2187	0.076*	
C6	0.56142 (15)	−0.1273 (2)	0.17356 (7)	0.0606 (5)	
H6	0.5959	−0.2274	0.1728	0.073*	
C7	0.57882 (12)	0.0055 (2)	0.14681 (6)	0.0458 (4)	
H7	0.6244	−0.0072	0.1280	0.055*	
C8	0.64575 (11)	0.40377 (18)	0.14754 (5)	0.0364 (3)	
C9	0.67094 (12)	0.4062 (2)	0.19461 (6)	0.0428 (4)	
H9	0.6339	0.3416	0.2108	0.051*	
C10	0.75135 (13)	0.5046 (2)	0.21792 (7)	0.0546 (5)	
H10	0.7665	0.5063	0.2495	0.065*	
C11	0.80791 (14)	0.5984 (2)	0.19495 (8)	0.0640 (6)	
H11	0.8616	0.6630	0.2107	0.077*	
C12	0.78427 (14)	0.5961 (3)	0.14796 (9)	0.0674 (6)	
H12	0.8224	0.6593	0.1320	0.081*	
C13	0.70373 (13)	0.4996 (2)	0.12451 (7)	0.0517 (4)	
H13	0.6885	0.4992	0.0929	0.062*	
C14	0.46541 (11)	0.42749 (19)	0.10506 (5)	0.0381 (3)	
H14A	0.4463	0.4708	0.1320	0.046*	
H14B	0.4889	0.5207	0.0897	0.046*	
C15	0.37153 (11)	0.35434 (19)	0.07395 (5)	0.0395 (3)	
C16	0.27485 (13)	0.3698 (2)	0.08237 (6)	0.0511 (4)	
H16	0.2649	0.4206	0.1087	0.061*	
C17	0.19352 (14)	0.3071 (3)	0.05024 (8)	0.0628 (5)	
H17	0.1277	0.3158	0.0548	0.075*	
C18	0.20984 (15)	0.2327 (3)	0.01190 (7)	0.0642 (5)	
H18	0.1552	0.1917	−0.0098	0.077*	
C19	0.30793 (15)	0.2184 (2)	0.00548 (6)	0.0542 (5)	
C20	0.3320 (2)	0.1393 (3)	−0.03588 (7)	0.0780 (7)	
H20A	0.3601	0.2215	−0.0527	0.117*	
H20B	0.2708	0.0944	−0.0546	0.117*	
H20C	0.3806	0.0512	−0.0268	0.117*	
C21	0.06735 (10)	0.80270 (18)	0.12103 (5)	0.0333 (3)	
C22	0.00363 (11)	0.90017 (18)	0.14826 (5)	0.0356 (3)	
C23	−0.07527 (13)	1.0013 (2)	0.12473 (7)	0.0519 (4)	
H23	−0.0884	1.0060	0.0932	0.062*	
C24	−0.13383 (15)	1.0943 (3)	0.14768 (9)	0.0668 (6)	
H24	−0.1863	1.1604	0.1316	0.080*	
C25	−0.11486 (15)	1.0896 (3)	0.19424 (8)	0.0659 (6)	
H25	−0.1544	1.1524	0.2097	0.079*	
C26	−0.03709 (15)	0.9914 (3)	0.21801 (7)	0.0578 (5)	
H26	−0.0241	0.9879	0.2495	0.069*	
C27	0.02182 (12)	0.8979 (2)	0.19507 (6)	0.0424 (4)	
H27	0.0744	0.8326	0.2114	0.051*	
C28	0.12469 (11)	0.65852 (18)	0.14885 (5)	0.0350 (3)	
C29	0.07775 (13)	0.5045 (2)	0.14868 (6)	0.0471 (4)	
H29	0.0135	0.4876	0.1302	0.057*	
C30	0.12589 (15)	0.3745 (2)	0.17595 (7)	0.0566 (5)	
H30	0.0939	0.2716	0.1754	0.068*	
C31	0.22188 (17)	0.3991 (3)	0.20394 (7)	0.0643 (6)	
H31	0.2533	0.3132	0.2226	0.077*	
C32	0.26986 (14)	0.5481 (3)	0.20410 (6)	0.0576 (5)	
H32	0.3346	0.5633	0.2224	0.069*	
C33	0.22213 (12)	0.6781 (2)	0.17688 (6)	0.0453 (4)	
H33	0.2555	0.7798	0.1773	0.054*	
C34	0.14015 (11)	0.92662 (19)	0.10486 (5)	0.0382 (3)	
H34A	0.1001	1.0187	0.0895	0.046*	
H34B	0.1868	0.9716	0.1313	0.046*	
C35	0.20135 (12)	0.85435 (19)	0.07364 (5)	0.0396 (3)	
C36	0.30642 (13)	0.8702 (2)	0.08174 (6)	0.0502 (4)	
H36	0.3428	0.9219	0.1079	0.060*	
C37	0.35558 (15)	0.8070 (3)	0.04974 (7)	0.0596 (5)	
H37	0.4260	0.8155	0.0542	0.072*	
C38	0.29968 (16)	0.7313 (3)	0.01125 (7)	0.0600 (5)	
H38	0.3320	0.6899	−0.0106	0.072*	
C39	0.19560 (14)	0.7175 (2)	0.00542 (6)	0.0482 (4)	
C40	0.12872 (14)	0.6369 (3)	−0.03596 (6)	0.0563 (5)	
H40A	0.0893	0.5496	−0.0265	0.084*	
H40B	0.1707	0.5909	−0.0548	0.084*	
H40C	0.0838	0.7190	−0.0528	0.084*	
N1	0.38751 (11)	0.28000 (18)	0.03621 (5)	0.0465 (3)	
N2	0.14697 (11)	0.77822 (18)	0.03610 (4)	0.0443 (3)	
O1	0.58575 (9)	0.24349 (15)	0.08070 (4)	0.0458 (3)	
H1A	0.5263 (15)	0.224 (2)	0.0571 (7)	0.055*	
O2	−0.00304 (8)	0.73711 (15)	0.08224 (4)	0.0461 (3)	
H2A	0.0333 (14)	0.704 (2)	0.0597 (7)	0.055*	

### Atomic displacement parameters (Å^2^)

**Table:**

	*U*^11^	*U*^22^	*U*^33^	*U*^12^	*U*^13^	*U*^23^
C1	0.0323 (7)	0.0332 (8)	0.0356 (8)	0.0010 (6)	0.0103 (6)	−0.0029 (6)
C2	0.0291 (7)	0.0338 (8)	0.0351 (7)	−0.0038 (6)	0.0027 (6)	−0.0047 (6)
C3	0.0417 (8)	0.0466 (9)	0.0436 (9)	−0.0030 (7)	0.0146 (7)	−0.0015 (7)
C4	0.0530 (10)	0.0646 (12)	0.0495 (10)	−0.0135 (9)	0.0148 (8)	0.0080 (9)
C5	0.0647 (12)	0.0560 (12)	0.0610 (12)	−0.0183 (10)	−0.0023 (10)	0.0204 (9)
C6	0.0569 (11)	0.0378 (10)	0.0798 (14)	0.0043 (8)	−0.0008 (10)	0.0102 (9)
C7	0.0401 (8)	0.0391 (9)	0.0561 (10)	0.0027 (7)	0.0065 (7)	−0.0017 (8)
C8	0.0307 (7)	0.0302 (8)	0.0489 (9)	0.0009 (6)	0.0102 (6)	−0.0020 (6)
C9	0.0385 (8)	0.0392 (9)	0.0494 (9)	−0.0045 (7)	0.0068 (7)	−0.0033 (7)
C10	0.0436 (9)	0.0513 (11)	0.0611 (11)	−0.0020 (8)	−0.0049 (8)	−0.0105 (9)
C11	0.0385 (9)	0.0465 (11)	0.0987 (17)	−0.0098 (8)	−0.0023 (10)	−0.0058 (11)
C12	0.0469 (10)	0.0524 (12)	0.1050 (18)	−0.0139 (9)	0.0211 (11)	0.0117 (11)
C13	0.0443 (9)	0.0479 (10)	0.0662 (11)	−0.0051 (8)	0.0193 (8)	0.0040 (9)
C14	0.0393 (8)	0.0325 (8)	0.0418 (8)	0.0032 (6)	0.0075 (6)	−0.0001 (6)
C15	0.0403 (8)	0.0361 (8)	0.0392 (8)	0.0003 (7)	0.0028 (6)	0.0067 (7)
C16	0.0428 (9)	0.0538 (11)	0.0550 (11)	0.0043 (8)	0.0069 (8)	0.0088 (8)
C17	0.0388 (9)	0.0658 (13)	0.0780 (14)	0.0004 (9)	0.0004 (9)	0.0173 (11)
C18	0.0563 (12)	0.0600 (12)	0.0630 (13)	−0.0040 (9)	−0.0149 (10)	0.0084 (10)
C19	0.0618 (11)	0.0499 (10)	0.0410 (9)	−0.0058 (8)	−0.0094 (8)	0.0105 (8)
C20	0.0978 (17)	0.0816 (16)	0.0469 (11)	−0.0147 (13)	0.0000 (11)	−0.0097 (11)
C21	0.0297 (7)	0.0333 (8)	0.0354 (8)	−0.0033 (6)	0.0039 (6)	−0.0029 (6)
C22	0.0314 (7)	0.0322 (8)	0.0436 (8)	−0.0046 (6)	0.0089 (6)	−0.0031 (6)
C23	0.0426 (9)	0.0504 (10)	0.0614 (11)	0.0094 (8)	0.0088 (8)	0.0026 (9)
C24	0.0529 (11)	0.0522 (11)	0.0984 (17)	0.0163 (9)	0.0234 (11)	0.0044 (11)
C25	0.0562 (11)	0.0584 (12)	0.0924 (16)	0.0027 (10)	0.0364 (11)	−0.0182 (11)
C26	0.0574 (11)	0.0637 (12)	0.0583 (11)	−0.0056 (9)	0.0255 (9)	−0.0131 (10)
C27	0.0399 (8)	0.0429 (9)	0.0448 (9)	−0.0023 (7)	0.0107 (7)	−0.0035 (7)
C28	0.0368 (7)	0.0336 (8)	0.0365 (8)	0.0025 (6)	0.0126 (6)	−0.0010 (6)
C29	0.0468 (9)	0.0380 (9)	0.0607 (11)	−0.0035 (7)	0.0208 (8)	−0.0030 (8)
C30	0.0602 (11)	0.0348 (9)	0.0875 (14)	0.0008 (8)	0.0433 (11)	0.0106 (9)
C31	0.0749 (13)	0.0558 (12)	0.0683 (13)	0.0251 (10)	0.0294 (11)	0.0240 (10)
C32	0.0510 (10)	0.0640 (12)	0.0543 (11)	0.0131 (9)	0.0043 (8)	0.0124 (9)
C33	0.0422 (9)	0.0409 (9)	0.0497 (10)	0.0003 (7)	0.0039 (7)	0.0012 (7)
C34	0.0397 (8)	0.0325 (8)	0.0428 (8)	−0.0022 (6)	0.0100 (6)	0.0030 (6)
C35	0.0454 (8)	0.0351 (8)	0.0398 (8)	0.0023 (7)	0.0129 (7)	0.0083 (7)
C36	0.0449 (9)	0.0533 (10)	0.0542 (10)	−0.0025 (8)	0.0150 (8)	0.0075 (8)
C37	0.0516 (10)	0.0627 (12)	0.0719 (13)	0.0062 (9)	0.0295 (10)	0.0160 (10)
C38	0.0701 (13)	0.0600 (12)	0.0604 (12)	0.0077 (10)	0.0368 (10)	0.0079 (10)
C39	0.0636 (11)	0.0471 (10)	0.0393 (9)	0.0075 (8)	0.0228 (8)	0.0078 (7)
C40	0.0629 (11)	0.0618 (12)	0.0479 (10)	0.0086 (9)	0.0201 (9)	−0.0079 (9)
N1	0.0483 (8)	0.0476 (8)	0.0400 (8)	−0.0026 (6)	0.0021 (6)	0.0045 (6)
N2	0.0500 (8)	0.0452 (8)	0.0385 (7)	0.0026 (6)	0.0118 (6)	0.0039 (6)
O1	0.0480 (6)	0.0527 (7)	0.0404 (6)	0.0015 (5)	0.0181 (5)	−0.0076 (5)
O2	0.0388 (6)	0.0544 (7)	0.0406 (6)	−0.0086 (5)	−0.0007 (5)	−0.0098 (5)

### Geometric parameters (Å, °)

**Table:**

C1—O1	1.4286 (17)	C21—C22	1.529 (2)
C1—C2	1.525 (2)	C21—C28	1.533 (2)
C1—C8	1.538 (2)	C21—C34	1.550 (2)
C1—C14	1.553 (2)	C22—C27	1.381 (2)
C2—C7	1.387 (2)	C22—C23	1.399 (2)
C2—C3	1.393 (2)	C23—C24	1.379 (3)
C3—C4	1.388 (2)	C23—H23	0.9300
C3—H3	0.9300	C24—C25	1.373 (3)
C4—C5	1.375 (3)	C24—H24	0.9300
C4—H4	0.9300	C25—C26	1.378 (3)
C5—C6	1.363 (3)	C25—H25	0.9300
C5—H5	0.9300	C26—C27	1.385 (2)
C6—C7	1.389 (3)	C26—H26	0.9300
C6—H6	0.9300	C27—H27	0.9300
C7—H7	0.9300	C28—C29	1.387 (2)
C8—C9	1.387 (2)	C28—C33	1.403 (2)
C8—C13	1.388 (2)	C29—C30	1.396 (3)
C9—C10	1.398 (2)	C29—H29	0.9300
C9—H9	0.9300	C30—C31	1.392 (3)
C10—C11	1.365 (3)	C30—H30	0.9300
C10—H10	0.9300	C31—C32	1.358 (3)
C11—C12	1.384 (3)	C31—H31	0.9300
C11—H11	0.9300	C32—C33	1.394 (2)
C12—C13	1.391 (3)	C32—H32	0.9300
C12—H12	0.9300	C33—H33	0.9300
C13—H13	0.9300	C34—C35	1.501 (2)
C14—C15	1.515 (2)	C34—H34A	0.9700
C14—H14A	0.9700	C34—H34B	0.9700
C14—H14B	0.9700	C35—N2	1.351 (2)
C15—N1	1.346 (2)	C35—C36	1.387 (2)
C15—C16	1.387 (2)	C36—C37	1.384 (3)
C16—C17	1.386 (3)	C36—H36	0.9300
C16—H16	0.9300	C37—C38	1.377 (3)
C17—C18	1.364 (3)	C37—H37	0.9300
C17—H17	0.9300	C38—C39	1.377 (3)
C18—C19	1.382 (3)	C38—H38	0.9300
C18—H18	0.9300	C39—N2	1.340 (2)
C19—N1	1.346 (2)	C39—C40	1.514 (3)
C19—C20	1.500 (3)	C40—H40A	0.9600
C20—H20A	0.9600	C40—H40B	0.9600
C20—H20B	0.9600	C40—H40C	0.9600
C20—H20C	0.9600	O1—H1A	0.96 (2)
C21—O2	1.4334 (17)	O2—H2A	0.96 (2)
			
O1—C1—C2	109.66 (11)	C22—C21—C28	111.41 (12)
O1—C1—C8	106.55 (11)	O2—C21—C34	109.08 (12)
C2—C1—C8	111.07 (12)	C22—C21—C34	108.18 (11)
O1—C1—C14	108.72 (12)	C28—C21—C34	112.34 (12)
C2—C1—C14	113.37 (12)	C27—C22—C23	118.02 (15)
C8—C1—C14	107.20 (12)	C27—C22—C21	123.47 (14)
C7—C2—C3	117.55 (15)	C23—C22—C21	118.48 (14)
C7—C2—C1	119.86 (13)	C24—C23—C22	120.83 (18)
C3—C2—C1	122.52 (14)	C24—C23—H23	119.6
C4—C3—C2	120.90 (16)	C22—C23—H23	119.6
C4—C3—H3	119.5	C25—C24—C23	120.24 (19)
C2—C3—H3	119.5	C25—C24—H24	119.9
C5—C4—C3	120.25 (17)	C23—C24—H24	119.9
C5—C4—H4	119.9	C24—C25—C26	119.75 (17)
C3—C4—H4	119.9	C24—C25—H25	120.1
C6—C5—C4	119.74 (17)	C26—C25—H25	120.1
C6—C5—H5	120.1	C25—C26—C27	120.15 (19)
C4—C5—H5	120.1	C25—C26—H26	119.9
C5—C6—C7	120.37 (18)	C27—C26—H26	119.9
C5—C6—H6	119.8	C22—C27—C26	121.00 (16)
C7—C6—H6	119.8	C22—C27—H27	119.5
C2—C7—C6	121.17 (16)	C26—C27—H27	119.5
C2—C7—H7	119.4	C29—C28—C33	117.78 (15)
C6—C7—H7	119.4	C29—C28—C21	119.76 (14)
C9—C8—C13	118.06 (15)	C33—C28—C21	122.37 (13)
C9—C8—C1	122.85 (13)	C28—C29—C30	120.78 (17)
C13—C8—C1	119.07 (14)	C28—C29—H29	119.6
C8—C9—C10	120.65 (16)	C30—C29—H29	119.6
C8—C9—H9	119.7	C31—C30—C29	119.95 (17)
C10—C9—H9	119.7	C31—C30—H30	120.0
C11—C10—C9	120.83 (19)	C29—C30—H30	120.0
C11—C10—H10	119.6	C32—C31—C30	120.22 (17)
C9—C10—H10	119.6	C32—C31—H31	119.9
C10—C11—C12	119.17 (17)	C30—C31—H31	119.9
C10—C11—H11	120.4	C31—C32—C33	120.01 (18)
C12—C11—H11	120.4	C31—C32—H32	120.0
C11—C12—C13	120.33 (18)	C33—C32—H32	120.0
C11—C12—H12	119.8	C32—C33—C28	121.25 (16)
C13—C12—H12	119.8	C32—C33—H33	119.4
C8—C13—C12	120.95 (18)	C28—C33—H33	119.4
C8—C13—H13	119.5	C35—C34—C21	115.16 (12)
C12—C13—H13	119.5	C35—C34—H34A	108.5
C15—C14—C1	114.95 (12)	C21—C34—H34A	108.5
C15—C14—H14A	108.5	C35—C34—H34B	108.5
C1—C14—H14A	108.5	C21—C34—H34B	108.5
C15—C14—H14B	108.5	H34A—C34—H34B	107.5
C1—C14—H14B	108.5	N2—C35—C36	122.03 (15)
H14A—C14—H14B	107.5	N2—C35—C34	115.57 (13)
N1—C15—C16	121.93 (15)	C36—C35—C34	122.35 (15)
N1—C15—C14	115.37 (13)	C37—C36—C35	118.10 (18)
C16—C15—C14	122.62 (15)	C37—C36—H36	121.0
C17—C16—C15	117.81 (18)	C35—C36—H36	121.0
C17—C16—H16	121.1	C38—C37—C36	119.67 (18)
C15—C16—H16	121.1	C38—C37—H37	120.2
C18—C17—C16	120.15 (19)	C36—C37—H37	120.2
C18—C17—H17	119.9	C37—C38—C39	119.50 (17)
C16—C17—H17	119.9	C37—C38—H38	120.2
C17—C18—C19	119.70 (18)	C39—C38—H38	120.2
C17—C18—H18	120.2	N2—C39—C38	121.49 (18)
C19—C18—H18	120.2	N2—C39—C40	115.73 (16)
N1—C19—C18	120.77 (18)	C38—C39—C40	122.77 (16)
N1—C19—C20	116.34 (18)	C39—C40—H40A	109.5
C18—C19—C20	122.87 (18)	C39—C40—H40B	109.5
C19—C20—H20A	109.5	H40A—C40—H40B	109.5
C19—C20—H20B	109.5	C39—C40—H40C	109.5
H20A—C20—H20B	109.5	H40A—C40—H40C	109.5
C19—C20—H20C	109.5	H40B—C40—H40C	109.5
H20A—C20—H20C	109.5	C19—N1—C15	119.63 (16)
H20B—C20—H20C	109.5	C39—N2—C35	119.21 (15)
O2—C21—C22	106.24 (11)	C1—O1—H1A	109.3 (11)
O2—C21—C28	109.39 (12)	C21—O2—H2A	109.5 (11)

### Hydrogen-bond geometry (Å, °)

**Table:**

*D*—H···*A*	*D*—H	H···*A*	*D*···*A*	*D*—H···*A*
O1—H1A···N1	0.96 (2)	1.89 (2)	2.724 (2)	144 (2)
O2—H2A···N2	0.96 (2)	1.92 (2)	2.718 (2)	139 (2)
C18—H18···O2^i^	0.93	2.70	3.518 (2)	147
C38—H38···O1^ii^	0.93	2.65	3.472 (2)	147

Symmetry codes: (i) −*x*, −*y*+1, −*z*; (ii) −*x*+1, −*y*+1, −*z*.
